# Outcome of Home-Based Early Intervention for Autism in Sri Lanka: Follow-Up of a Cohort and Comparison with a Nonintervention Group

**DOI:** 10.1155/2016/3284087

**Published:** 2016-06-22

**Authors:** Hemamali Perera, Kamal Chandima Jeewandara, Sudarshi Seneviratne, Chandima Guruge

**Affiliations:** ^1^University of Colombo, Colombo, Sri Lanka; ^2^Department of Family Medicine, Faculty of Medical Sciences, University of Sri Jayewardenepura, Colombo, Sri Lanka; ^3^Faculty of Medicine, University of Colombo, Colombo, Sri Lanka; ^4^National Hospital, Colombo, Sri Lanka

## Abstract

This paper presents the outcome of a home-based autism intervention program (HBAIP) in 18- to 40-month-old children newly diagnosed and treatment naïve. Intervention was exclusively implemented at home. Outcome was measured at 3 months and 6 months after intervention and compared with a group of newly diagnosed children with autism who were >40 months at intake but had not received any autism specific clinical management. Aim was also to estimate whether natural development would contribute to gain in skills and compare with the effect of intervention. Five selected parameters of behavior representing social interaction and social communication were used to assess outcome. Results showed a statistically significant improvement between preintervention and postintervention in all the measured parameters. The effect size was large when compared to preintervention and gains were indicated by changes in mean scores and *p* values within a narrow confidence interval. Highest gains were in first 3 months of postintervention which continued up to 6 months. Although the comparison group was more advanced in the measured skills at intake, they were significantly below the level reached by experimental group at 3 months and 6 months after intervention. This study was registered in the Sri Lanka Clinical Trials Registry (SLCTR/2009/011).

## 1. Introduction

Autism is a complex neurodevelopmental disorder characterized by impairment in social behavior and communication, along with a restricted repertoire of activities and interests [1]. Many explanations are given regarding the underlying cognitive and affective deficits that cause such development deviance and impairment in children with autism. These in turn are used in developing methods for interventions. In consequence, a range of intervention programs have been developed over the past few decades, with some of them claiming to be more effective than others. Behavioral methods are the most widely used interventions in children with autism. Intensively implemented intervention programs such as Applied Behavioral Analysis (ABA) have been shown to significantly improve functioning in children with autism [2, 3]. Such intensive interventions are broadly termed Early and Intensive Behavioral Intervention (EIBI) and are known to improve preacademic skills, language, and social skills and reduce stereotypies and self-injury [4, 5]. However, there is considerable individual variation in outcome of different EIBI programs [2, 4, 6]. Reviews of EIBI have not always shown evidence for efficacy in all cases [6]. Also, reliability in many outcome studies on intervention programs for autism is hampered by methodological flaws and small sample sizes [6–10].

A basic deficit in children with autism is the lack of understanding of initiating and responding to joint attention. Joint attention by definition is visually coordinating attention to an event or object with another individual, sharing interest and social engagement, and showing an understanding that the partner is sharing the same focus [11]. Deficit in joint attention and symbolic play skills have been identified as two specific social interactional and communication difficulties in children with autism [11, 12]. These deficits are also considered as early predictors of autism and can be recognized before the age of one year [13]. Targeting joint attention provides a clear direction for intervention [14]. Hence, intervention to improve joint attention together with the closely associated symbolic play skills will have a major impact on the social functioning and language development of children with autism. Interventions that target deficits in joint attention skills use face to face social play with an adult [11–17]. The Early Start Denver Model (ESDM) emphasizes joint attention through socially oriented activities and building play skills [18, 19]. ESDM also uses teaching within family routines as a component of the program [18–20].

Efficacy of most models of intervention has been studied in resource-rich specialist settings. Most studies have focused on home-based training with parent involvement, as an adjunct to a specialist centre based intervention [6, 7, 18, 21, 22]. The main objective of our study was to measure the outcome of a home-based autism intervention program (HBAIP) for 18- to 40-month-old children, where the intervention was exclusively implemented at home. Such a program was justified for several reasons. Firstly, in Sri Lanka at present, there are no state sponsored health programs in the community for autism. A few private sector facilities are available, but, for majority of families, these remain inaccessible because of high cost. Secondly, multidisciplinary resources (speech therapists, occupational therapists, and psychologists) were not readily available at the tertiary care pediatric hospital Child Mental Health Unit (CMHU) where this study was conducted. Thirdly, the known prevalence of autism in Sri Lanka is 1 in 93 [23]. Although a rising prevalence is reported from other parts of the world, more recent epidemiological data is not available. Under the circumstances, the option of a home-based program was adopted to ensure that after diagnosis intervention for the child commenced without delay or interruption. A minor objective of the study was to answer the following question: “If children with autism did not receive intervention, would they have improved in skills nevertheless, due to natural development?” Hence, the data at intake and after intervention of the experimental group was compared with data at intake of a group that did not receive autism specific intervention until after 40 months.

## 2. Method

### 2.1. Study Design

This was a prospective intervention study.

### 2.2. Experimental Sample

Consecutive children aged 18 to 40 months and newly registered to the HBAIP over a period of one year who fulfilled the inclusion criteria were recruited to the study. All children included in the experimental sample were diagnosed with autism for the first time at intake and had not received developmental interventions of any form previously. Children excluded from the study were (i) those diagnosed with other pervasive developmental disorders and Asperger disorder, (ii) those with severe cognitive impairment with autistic features, due to the difficulty in establishing a clear primary diagnosis, (iii) those diagnosed with autism having associated motor and sensory disorders and genetic disorders, to avoid any confounder bias, (iv) those who had received other developmental interventions before intake and during the course of the study, and (v) those who dropped out before completion of the intervention period.

### 2.3. Comparison Sample

The comparison sample comprised children over the age of 40 months who newly registered to HBAIP. They too received the diagnosis of autism for the first time at intake. The exclusion criteria (i), (ii), and (iii) used in the selection of the experimental sample were applied to the comparison sample as well. They had received nonspecific developmental interventions before intake and some were attending preschool.

### 2.4. Diagnosis

In all children, the diagnosis of autism was made clinically using DSM IV TR criteria [1]. The diagnostic procedure involved a comprehensive interview with parents and observation of the child's behavior in the clinical setting. In addition, Childhood Autism Rating Scale (CARS) was used to establish the severity of autism. A senior clinician in child and adolescent psychiatry carried out the diagnostic procedure.

### 2.5. Home-Based Intervention Process

The mothers were expected to provide one to one, face to face play activity with the children, 20 to 30 minutes at a time, 2 hours a day. Emphasis was made to schedule the therapy times in advance into the daily routine at home to minimize intrusion from other activities. Any other adults in the household such as grandparents, older siblings, or a nanny were also encouraged to join in. The activities to be carried out at home were demonstrated to the parents, which were supported with written material and video clips on how to play with the child. During the scheduled play time the mother shared any activity the child had initiated. The mother continuously talked to the child in simple clear speech and made physical contact as appropriate while playing. The mother also initiated activities some of the time with available play material to get the child's attention and facilitate symbolic playing. The aim was to encourage joint attention with the child to promote sustained eye contact, sharing, pointing and requesting, imitating, and showing response when called by name. In addition to the structured play activities, parents were encouraged to facilitate joint attention promoting activities during daily routines such as meal times. Although certain activities were demonstrated to parents at the beginning, they were encouraged to use a wide range of activities and to be flexible in using familiar material available at home in working with the child.

The child and the parents were reviewed once a month to provide further support and guidance regarding difficulties faced in implementing the activities and to discuss other possible play options. The total time spent in educating and training mothers was about 7 hours during the study. A senior clinician assisted by 3 junior clinicians with over 5 years of experience with autism was associated in the training of mothers.

### 2.6. Preintervention and Postintervention Assessment

Preintervention assessment was carried out in both experimental and comparison groups. Five independent parameters that represent social interaction and social communication in the child were used to measure the outcome of intervention. The basis for selection of these measures was their relevance to social interaction and communication and being readily understood by the mother and demonstrable to the child. At preintervention, 5 measures were assessed by asking the following questions: (i) Does the child give sustained eye contact? (ii) Does the child socially reciprocate an action by the mother? (iii) Does the child imitate a simple action the mother demonstrates? (iv) Does the child respond when mother calls by name? (v) Does the child point to request something from mother? In the clinical setting, the mother engaged and interacted with the child in order to demonstrate the presence or absence of the skill. A score was given on joint agreement between the mother and an independent assessor on the estimated level of skill in the child for each measure by plotting on a visual analogue scale of 0 to 100, with 0 being total absence of the skill and 100 being present every time it was tested. The independent assessor was not involved in the diagnostic process or training of the mother. Freedom of choice on technique for demonstrating the presence of the skills in the child as well as several trials was given to the mother in assessing each measure. This was the only assessment carried out in the comparison group.

Postintervention assessment was made in the children of the experimental group using exactly the same procedure at the completion of 3 months and 6 months from commencement of intervention. Here too, a score was given on joint agreement between parent and the independent assessor on the estimated level of skill in the child for each measure by plotting on a visual analogue scale of 0 to 100. All assessments were made at the CMHU.

The data were analyzed using statistical software program SPSS version 16. Statistical methods used were frequency distribution, comparison of means with Student's *t*-test, and the effect size using an accepted formula. Since the data was not normally distributed, “unstandardized” mean difference was used in calculating the effect size.

Effect size = “unstandardized” mean difference [(mean at 3 or 6 months) − (mean at 0 or 3 months)]/standard deviation.

The level of statistical significance for this study was set at *p* <  0.05.

Approval for the study was obtained from the Ethical Review Committee, Faculty of Medicine, University of Colombo, Sri Lanka.

## 3. Results

### 3.1. Experimental Sample

A total of 62 children, with 18 to 40 months of age (mean: 32 months, SD: 7.53), participated in the study. Of them, 26 (41.9%) were below 30 months of age and 48 (77.4%) were male. None of the children received a change of diagnosis or an additional diagnosis of developmental or any other disorder during the period of the study. On CARS, all children were rated as severe autism with a mean score of 45.24 (range: 39–50, SD: 3.45).

The commonest presenting problem was delayed language development in 35 (56.5%) with social and language regression in the second year in 10 (16.1%). Medical comorbidity was present in 13 (20.9%) with epilepsy in 6 (9.7%). Valid information was not available on the presence of autism in siblings or any other first-degree relatives.

### 3.2. Characteristics of Parents of Children in the Experimental Group

The mean age of the mothers was 33 years (range: 23–44 years, SD: 5.00) and that of the fathers 37 years (range: 25–47 years, SD: 4.72). Thirty-two (51.6%) mothers and 62 (100%) fathers were in full-time employment. Of the mothers in full-time employment, 16 (25.8%) stopped working temporarily to provide the prescribed intervention for the child. Regarding education of the parents, 56 (90.3%) mothers and 52 (83.8%) fathers had school based education up to the age of 16 to 18 years. Moreover, 6 (9.7%) mothers and 10 (16.1%) fathers had a university degree. Other family members made major contributions in the intervention for 26 (41.9%) of mothers. The index child was the only child of the parents in 39 (62.9%) cases.

### 3.3. Comparison Sample

A total of 42 children, with 43 to 70 months of age (mean: 54.2 months, SD: 12.9), were included in the comparison sample. Of them, 32 (76.2%) were male. On the CARS, the mean score was 40.74 (range: 33 to 50, SD: 5.85). Of them, 8 (19%) received a score less than 35 indicating mild-to-moderate autism. The remaining 34 (81%) were placed in the category of severe autism.

The commonest presenting complaint was poor language development, which was in 36 (85.2%). Medical comorbidity was present in 9 (19.5%) with epilepsy in 2 (4.8%). All were attending mainstream preschool or school and some had developmental intervention such as speech therapy.

Mean assessment scores at intake for the experimental group (*n* = 62) and the comparison group (*n* = 42) in the 5 domains of measurement are given in Table 1. Also, Table 1 gives the change of mean scores at 3 and 6 months following intervention in the experimental group.

In Table 2, the statistical significance of mean difference in assessment scores between the study and comparison groups is given. This comparison is made at preintervention and at 3 months and 6 months of intervention.

## 4. Discussion

At baseline, all children in the experimental group fell into the category of severe autism on CARS. Following intervention, the results showed a statistically significant improvement in all the parameters that were measured (Tables 1 and 2). The effect size was high in all domains of skills when compared to preintervention level (Table 3). In addition, the significant gains were indicated by changes in mean scores and *p* value within a narrow confidence interval (Tables 1, 2, and 3). Although the highest gains were in the first 3 months after intervention, significant increase in measures continued up to 6 months. The largest effect size and change in mean scores were noted in the improvement of eye contact and the lowest was in pointing behavior, though both outcomes were highly significant (Tables 2 and 3). The outcomes indicate that the intervention strategies used in the study were highly effective and that improvement was rapid.

When the experimental group was compared with the comparison group, the level of skills at intake as measured in the 5 domains was more advanced in the latter, which was statistically significant (Table 2). This was evident in all measurements except imitative behavior (Table 2). This indicates that some developmental gains had taken place in the comparison group, without receiving any autism specific intervention. Despite these gains, the majority (81%) was still having severe autism when rated on CARS. Also, the comparison group had not reached the targets achieved by the experimental group during the years when they had no intervention or received the nonspecific interventions. These findings indicate that while some natural developmental processes may have taken place in these children, specific measures used in this study provided a better outcome. It is known that intervention for autism has more favorable outcome over time when compared to nonintervention [24, 25].

The success of the HBAIP is attributable to child, parent, and training related factors. Firstly, almost 42% of the intervention group commenced intervention before 30 months of age. Hence, early intervention may have been responsible for the favorable outcome, as shown in other studies too [26]. Secondly, offering individualized time to parents, demonstration of activities to be carried out at home and written instructions, education about autism, allowing time to discuss problems, monthly follow-up, instructions given to suit the individual environment, and both child-centred and parent-centred approach are likely to have contributed to the success of the program. Although discrete skills were measured in evaluating outcome, broader learning targets were used at home. High level of motivation would have been an important parent related factor as indicated by 25% leaving employment to work with the child. Also, 62.9% being singleton would have allowed extra time to spend with the child. In addition, it is well accepted that parent training in autism treatment programs positively contributes to the outcome [7]. The parents in this study had the benefit of being educationally resourceful, which would have helped in better comprehension of autism and the prescribed intervention strategies. Besides, 41.9% received added support of the extended family. Thirdly, the trainers contributed to and facilitated the process through individualized training and ongoing support. Finally, all learning for the child happened in his natural environment with familiar people. Natural environment is more favored as the setting for intervention in young children and is more beneficial than specialist-centre based intervention [7, 22].

HBAIP was implemented exclusively by parents acting as full-time therapists. Most other studies on parent implemented intervention in autism have utilised other professional resources with parents acting as cotherapists [7]. Concurrent improvement in verbal language could not be assessed due to lack of objective measures as parents may not have differentiated between functional and nonfunctional words. Nevertheless, results of our study are comparable with other programs that have used similar strategies as their main form of intervention [25, 27]. Home-based programs have shown similar results in efficacy to those implemented in specialist therapeutic settings [7, 27].

The evaluation of outcome was scored by the parents as well as an independent assessor. Utilising an independent assessor was useful in reducing the bias of possible overestimation or underestimation of the level of skills in the child. Also, the possible negative impact of the less familiar hospital setting on the child's behavior would have been overcome to some extent by joint score with agreement between the mother and the independent assessor.

Certain shortcomings of the study need to be mentioned. This is not a randomized controlled study. The outcome was measured only up to 6 months. The sustenance of the improvement beyond this period or continuing progress cannot be predicted from this study. All children in the experimental group had severe autism on CARS, which could be seen as a selection bias. The exclusive use of parents in evaluating the outcome of intervention limited the domains of measurements that could be used. The parents' feedback was mostly related to child's behavior in the home and other familiar settings, which cannot be generalized to new contexts and contact with different persons. The evaluation of the outcome by the parents who themselves were therapists may have introduced bias but reduced to some extent by jointly agreed score with the independent evaluator. The comparison group was not age-matched with experimental group. However, to answer the question whether natural development will improve skills in autism, it was necessary to take a group that had already passed the age of the experimental group. Another weakness of the study was that parents' satisfaction with the program and their experience in the implementation were not objectively measured. Understanding this aspect is important if the program is to be introduced more broadly into clinical settings.

## 5. Conclusion

This study shows the efficacy, feasibility, and trainability of parents to carry out intervention at home in the role of a full-time therapist. The results of this study are valuable to Sri Lanka as they provide an effective direction in the management of young children with autism.

## Figures and Tables

**Table 1:** Mean scores for each domain of measurement at intake to the study and at 3 and 6 months after intervention for the experimental group.

Measure	Experimental group (*n* = 62) mean score (range, SD)	Comparison group (*n* = 42) mean score (range, SD)
*Sustained eye contact*		
At intake	2.02 (0–50, 7.15)	17.86 (0–60, 18.45)
Intervention		
At 3 months	52.98 (10–90, 23.91)	—
At 6 months	74.44 (10–95, 19.14)	—

*Response to name*		
At intake	0.56 (0–10, 2.24)	17.14 (0–80, 19.94)
Intervention		
At 3 months	48.87 (0–100, 27.87)	—
At 6 months	73.71 (5–95, 23.27)	—

*Social reciprocity*		
At intake	0.81 (0–10, 2.43)	15.24 (0–60, 18.27)
Intervention		
At 3 months	46.05 (0–100, 26.16)	—
At 6 months	72.42 (25–75, 21.93)	—

*Imitative behavior*		
At intake	0.81 (0–10, 1.23)	3.1 (0–40, 7.06)
Intervention		
At 3 months	47.58 (0–100, 28.99)	—
At 6 months	70.65 (25–75, 21.18)	—

*Pointing*		
At intake	0.32 (0–10, 1.78)	11.79 (0–60, 17.34)
Intervention		
At 3 months	35.89 (0–100, 27.55)	—
At 6 months	61.77 (15–85, 20.75)	—

Comparison group had only one set of data as given in Table 1.

**Table 2:** Statistical significance of mean difference in assessment scores in domains of measurement between experimental group (*n* = 62) and comparison group (*n* = 42).

Measure	Mean difference in assessment scores (95% CI)	*t*	*p*
*Sustained eye contact*			
Before intervention (at intake)	−15.76 (−20.60 to −10.91)	−6.45	<0.001
Intervention			
At 3 months	28.27 (19.02–37.51)	6.06	<0.001
At 6 months	50.53 (41.84–59.21)	11.53	<0.001

*Response to name*			
Before intervention (at intake)	−16.50 (−21.55 to −11.44)	−6.47	<0.001
Intervention			
At 3 months	26.72 (16.54–36.90)	5.52	<0.001
At 6 months	50.51 (40.98–60.05)	10.50	<0.001

*Social reciprocity*			
Before intervention (at intake)	−13.94 (−18.62 to −9.27)	−5.91	<0.001
Intervention			
At 3 months	24.44 (15.31–33.55)	5.31	<0.001
At 6 months	45.49 (37.62–53.34)	11.48	<0.001

*Imitative behavior*			
Before intervention (at intake)	−2.93 (−4.74 to −1.12)	−3.20	0.002
Intervention			
At 3 months	36.58 (28.50–44.65)	8.98	<0.001
At 6 months	57.30 (50.78–63.83)	17.42	<0.001

*Pointing*			
Before intervention (at intake)	−11.30 (−15.71 to −6.89)	−5.08	<0.001
Intervention			
At 3 months	18.38 (8.98–27.78)	3.88	<0.001
At 6 months	42.89 (33.90–51.88)	9.46	<0.001

95% CI: 95% confidence interval; *t*: paired *t* distribution; *p*: statistical significance at 0.05.

**Table 3:** Effect size for outcome in the experimental group at 3 months and 6 months after intervention and between 3 and 6 months.

Outcome measure	ES 0–3 months after intervention (95% CI)	ES for 0–6 months after intervention (95% CI)	ES for 3–6 months after intervention (95% CI)
Sustained eye contact	1.67 (1.27–2.09)	2.71 (2.65–2.76)	1.03 (0.97–1.08)
Response to name	1.50 (1.11–1.91)	2.46 (2.40–2.51)	0.95 (0.90–0.99)
Social reciprocity	1.54 (1.13–1.93)	2.51 (2.45–2.56)	0.87 (0.84–0.93)
Imitative behavior	1.46 (1.11–1.80)	2.23 (2.16–2.29)	0.75 (0.70–0.79)
Pointing	1.12 (1.07–1.16)	1.85 (1.79–1.90)	0.91 (0.86–0.95)

95% CI: 95% confidence interval; ES: effect size.

## Additional Points

The setting of the study is Lady Ridgeway Hospital for Children, Colombo, Sri Lanka.
